# Supplementary material for: Housing during the asylum process and its association with healthcare utilization for common mental disorders among refugees in Sweden: A nationwide cohort study
Source: PLOS Glob Public Health. 2025 May 28;5(5):e0003987. doi: 10.1371/journal.pgph.0003987 (PMC12118870; doi:10.1371/journal.pgph.0003987)
Supplement: Attachment — Submitted filename: Response to reviewers_CharlottavEggermontArwidson etal.docx [file pgph.0003987.s004.docx]

**Ref: Submission** **PGPH-D-24-01909**

**Response to Reviewers**

Dear Mr. Essex,

On behalf of my co-authors, I would like to thank you for giving us the opportunity to submit a revised draft of the manuscript "Housing during the asylum process and its association with healthcare utilization for common mental disorders among refugees in Sweden: A nationwide cohort study" to PLOS Global Public Health. We appreciate the time and effort that you and the reviewers dedicated to providing feedback on our manuscript and are grateful for the insightful comments to our paper. We have been able to incorporate most of the suggested changes made by the reviewers. The changes are highlighted within the manuscript by using tracked changes.

Please see below, in red, for a point-by-point response, to the reviewers’ comments and concerns.

**Comments from reviewer 1**

*Overall comments*

This is a very important contribution to existing literature. Thank you. Your analysis and/or discussion could be improved by few factors that were not covered in the analysis. Please see the attached document.

- Response: Thank you for your positive feedback and for your helpful comments. We have revised the manuscript accordingly and hope you now find it suitable for publication.

*Abstract*

Line 19: current mental health or future mental?

- Response: Thank you for pointing out the vagueness in our wording. We have revised it to clarify that it refers to current mental health. It now reads as follows: “Research also suggests that institutional accommodations during the asylum process might be more detrimental to their mental health while living in these settings compared to private accommodations”

Line 21: Use the full term of CDMS

- It has been revised

*Introduction*

Line 81-83: the evidence you have presented so far seems to clearly indicate that EBO is associated with positive outcomes, compared to ABO. So the sentence here is unclear. Why is there a need to explore further? IN other words, your justification needs to be bit more clearer. Is there a doubt that ABO might be positive or are the results mixed from previous studies ? if so, that is the justification for your study.

- Response: Thank you for your feedback. We appreciate your highlighting this point and recognize that we can be clearer in conveying our intended meaning. To clarify, we wish to emphasize that, to date, there are no studies comparing EBO and ABO in relation to health outcomes in Sweden. The only existing research in this area focuses on housing integration and employment. Therefore, from a health perspective within the Swedish context, there is a significant gap in knowledge regarding this topic. We have clarified this in the text that now reads as follows: “However, aside from studies comparing ABO and EBO concerning housing integration and employment, there are currently no studies in the Swedish context that specifically compare different housing types during the asylum period and their relation to different health outcomes. Hence, there remains a significant gap in knowledge regarding this issue.

*Method*

Line 143 please provide info on how many such instances were there and how this might impact your analysis

- Response: Thank you for raising this point. Unfortunately, no official statistics are available, and the Swedish Migration Agency has been reluctant to provide estimates. However, following this review, we revisited the possibility of a rough estimate. In recent personal communication, the Migration Agency estimated that around 10% of individuals change housing type today, though this figure doesn’t necessarily reflect the situation from 2010–2012. However, this approximation offers some insight, and it remains a limitation to our study which we have tried to clarify further under the section limitations. We have rephrased the paragraph under limitations, and it now reads as follows: “Another notable limitation is that up to 10% of the study population may have experienced both exposures, moving between ABO and EBO. While it is difficult to determine the reasons behind this changing of housing type and its potential association with mental health, it is important to interpret our findings with this consideration in mind”

Under the method section the paragraph now reads as follows: “However, an estimated 10% of individuals switch housing arrangements during the process—such as those residing in EBO transitioning to ABO, or vice versa, individuals living in ABO shifting to EBO (Swedish Migration Agency, personal communication, October 24, 2024).”

Line 148: Is there a difference in duration stay in EBO vs ABO i.e. time take to process the asylum application for individuals in ABO Vs. EBO?

- Response: Thank you for your question. We agree that it would have been valuable to compare the duration of stay based on different housing types. Unfortunately, the Swedish Migration Agency does not provide this type of data, and the dataset available for this study lacks information on the length of the asylum process for individuals in our sample, as well as any variables that would allow us to calculate it. However, since housing type, whether ABO or EBO, is not considered when processing asylum claims, any variation in processing times between ABO and EBO must stem from differences in other characteristics, with country of origin appearing to be one of the most significant factors. We have clarified this in the text and it now reads as follows: “The Swedish Migration Agency does not offer statistics on average processing times apart from a distribution based on applicants' citizenship. Additionally, the dataset available for this study lacks variables that would enable the calculation of individual processing times, which would allow for a comparison of length of stay based on housing type. However, there are no indications that processing times would differ, as the Swedish Migration Agency applies equal processing standards to individuals from both ABO and EBO”

*Results*

Line 237: From an intersectionality perspective, accommodation cannot be the only factor associated factor with poor mental health outcomes. It accommodation type interacting with other factors that is likely to result in poor mental health outcomes. In other words, the asylum seekers who to choose to stay in EBO should be different (on some characteristic) to the ones that chose to stay in ABO. People ending up in ABO or EBO is not a randomised selection/distribution i.e. there should be a difference between the groups However, your analysis here indicates that there are no differences in factors that you chose to consider here. This is very important to discuss further. Is it because that your data did not have other factors that might have been interesting to be considered here? If so, which are those factors? Could it be that people who chose ABO had limited social network or social capital compared to those who chose EBO? Social capital and social network are predictors of mental health Or what are the other potential explanations? It would be very important to discuss this in the discussion section.

- Response: Thank you for raising this. We certainly agree with you that there are multiple additional factors influencing mental health beyond accommodation. In addition, we would like to clarify that there are several differences between our two study groups with regard to sociodemographic factors that may be linked to poor mental health, which we have discussed in detail in the results section. In the association analyses presented in the manuscript (starting from Table 2), we adjusted for the factors listed in Table 1, which revealed that several of these factors were associated with higher risks for the two outcomes. However, an unexplained association with housing type persisted. The validity of this study, therefore, depends on whether potential differences between the ABO and EBO populations have been sufficiently addressed through these adjustments. We believe that the factors adjusted for (Table 1) in the association analysis represent key variables associated with mental health, thereby lending credibility to our findings. However, we acknowledge that including additional covariates could further strengthen the claims made in this study, and it is important to thoroughly discuss and reflect on these issues. Similarly, we agree that social network or social support may indeed be factors that differs between the two groups we are comparing and could, therefore, influence the association between housing and healthcare utilization. We have addressed this in the discussion and will further elaborate on this in that section. Furthermore, to clarify our findings, we have made a minor revision to the text at the beginning of the results section. It now reads as follows: “In terms of differences between the two groups regarding sex, level of education, and age there were only subtle variations. More notable differences…”

Line 242: were any further analysis conducted to see why refugees who lived in ABO , that had relatively better living standards than EBO, had negative health outcomes?

- Response: Thank you for the question. While it would have been interesting to explore further, we don't see it as fitting within the scope of this study. Furthermore, as outlined in the limitations section, there are certain constraints with the variable "disposable income," which may restrict the conclusions we can draw regarding its impact.

Line 245: Is there a reason why the OR was not adjusted for duration of stay in in ABO Vs. EBO as existing literature clearly shows that duration of stay (or any exposure for that matter) plays a major role in the (severity of the)outcome

- Response: We agree that it would have been valuable to adjust for the length or duration of the asylum process, as other literature highlights it as an important factor in mental health. However, as we mentioned earlier, we did not have access to that data. Furthermore, we believe that including it is unlikely to alter our main findings, since, as noted in our previous comment, there is no evidence suggesting that the waiting times for those living in ABO or EBO differ solely based on accommodation type. This has been outlined in the limitations and we hope this sufficiently addresses your concern.

*Discussion*

Line 294: will be good discuss why this might be the case. Is it because of better access to healthcare service in urban areas?

- Response: These results that you are highlighting are findings from the moderation analyses that suggest that urbanicity appears to influence ABO and EBO groups differently. Specifically, individuals from EBO in more urbanized areas tend to use more medication for CMD than those from ABO in urbanized areas. This suggests that the difference is influenced by factors beyond simply residing in an urban area and actual access to healthcare services at the time for measurement. It’s challenging to interpret the reasons behind this difference—and to address this we have added a sentence that reads as follows: “This may be challenging to interpret, but one possible explanation is that the EBO group has been more exposed to urban environments than the ABO group. People living in EBO more often reside in larger cities during the asylum period, which increase the likelihood of encountering factors associated with a higher risk of mental disorders [28].”

Line 303: you have cited one study only, not not 'studies'

- Response: Thanks for pointing this out. We have revised and it now states: “In addition, in a study…”

Line 314: this statement is contrary to what has been stated earlier. Page 3 "However, as national asylum systems differ in their policies and practices, leading to diverse living conditions for asylum seekers, it is important to acknowledge that research findings may not be directly transferable from one context to another."

- Response: Thank you for pointing out these seemingly contradictive statements. We have revised the text to avoid any appearance of contradiction, while also conveying that insights from other studies can still be valuable when contextual differences are taken into account. The first sentence at page 3 now reads as follows: “However, as national asylum systems sometimes differ substantially in their policies and practices…”

The other paragraph has also been rephrased and now states the following: “However, since refugees often share a pre-migration context, and assuming that the post-migration context does not significantly differ in policies and practices —an assumption that is often difficult to defend, given the substantial variation that typically exists between countries— the results may still provide some valuable insights for other contexts. Nonetheless, there is significant mobility in the policy area of asylum reception, and asylum policies within the EU are rapidly changing, affecting how consistent over time these results may be.”

Line 337: please add reference

- Response: Thanks for highlighting this. We have added a reference as well as a paragraph to support our statement. The paragraph now reads as follows: “The dispersal and no-choice policy is closely related to psychosocial factors, such as lack of control and autonomy [10], which we argue might constitute the most notable difference between private and institutional accommodations in Sweden. This is further supported by the study conducted with asylum seekers in EBO, which highlights that a significant factor in opting against ABO is the perceived loss of autonomy and control associated with not having the freedom to choose their living arrangements [21].”

Line 353: please see my earlier query on the absence of difference between the EBO and ABO groups

- Response: Thank you for raising this point. We appreciate your feedback and understand your concern that, despite our adjustments, there may be other predisposing factors beyond those at our disposal that could influence the results. In response, we have revised the wording to adopt a more nuanced description regarding the extent of our adjustments for sociodemographic factors. It now reads as follows: “However, as we adjusted in our analysis for sociodemographic factors such as age, sex, region of origin, children and level of education, which did not change our estimates, it is reasonable to assume that predisposing components have been accounted for to considerable extent”.

Line 364: however, you can discuss the results using existing literature . The discussion here is limited. Please my earlier comments regarding this

- Response: Thank you for highlighting this: We have added a paragraph that extends the discussion and reorganized the text for clarity, moving some content from the limitations section to the discussion. The last paragraph of the discussion now reads as follows: “In addition to recognizing social support and social networks as factors influencing healthcare utilization, it’s essential to acknowledge their impact on overall mental health and well-being. Various studies emphasize that social support is a crucial enabler of mental health among migrant groups, while a lack of social networks and support can negatively affect mental health [4, 35]. In the Swedish context, research on a comparable refugee population has underscored the importance of social support for general health [36], suggesting that variations in CMDs could partly stem from differences in available social support. Forced migration generally disrupts pre-existing networks and limits access to broader social ties [37, 38], yet the assumption that EBO individuals may choose this option due to having family or friends in Sweden suggests they might have greater access to social resources, which thereby could have an influence on mental health. Furthermore, living with family and friends in the community may offer a greater social network and social support, potentially serving as a buffer against mental ill-health. Given this, accounting for social support could have enhanced the robustness of our findings. However, this data is not accessible in Swedish registries, highlighting an area for future research. We therefore encourage longitudinal survey studies to further explore the role of social support in mental health outcomes for a more comprehensive understanding of the associations between housing and different mental health outcomes.”

The paragraph under limitations now reads: “Furthermore, as already mentioned, another source for residual confounding could be social support or social networks. In relation to our study, it is reasonable to consider that EBO and ABO might differ in terms of social support as outlined earlier. Accounting for the level of social support might have added depth to our analysis and explained part of the association between housing type and mental health service use.”

We have also added a section in the conclusion addressing the need for further research on the role of social support, among other factors, to emphasize its importance. The section reads: “To advance these discussions, future research should aim to develop a more comprehensive understanding of the links between housing and mental health for asylum seekers, with a particular focus on contextual factors and the role of social support. Such research should include both qualitative studies that provide in-depth insights into the lived experiences and challenges of asylum seekers as well as quantitative studies – ideally longitudinal surveys – to monitor the health outcomes over time and comparing the effects of different housing types. Ideally, these studies would incorporate a broad set of measures, including social support, pre-migration trauma, mental health status, and the duration of the asylum process.”

Line 368: please provide references that support this assumption. regarding which outcome? Your analysis shows that OR for poor mental health outcomes persists after a permit is granted. Unclear.

- Response: Thank you for your comment. We have reevaluated the sentence in question and agree that it may have been somewhat unclear. In response, we have opted to remove it to enhance the clarity of the text.

Line 402: as indicated in my earlier comments, this discussion needs to be expanded further. Would it be not possible to estimate the duration of asylum process by taking the difference between when the individual was granted residence permit minus when they registered at the accommodation ABO/EBO? Since social support, duration of stay (i.e length of asylum procedure) are key covariates that might seriously impact the statistical significance- these need to be discussed further

- Response: We appreciate your bringing these matters to our attention. We have addressed your concerns in various comments and revisions of the manuscript to provide the necessary clarifications. We hope that these adjustments and clarifications adequately meet the requirements you have identified.

**Comments from reviewer 2**

*Overall comments*

The authors have presented the results of a nationwide cohort study on housing during the asylum process and its association with healthcare utilization for common mental disorders among refugees in Sweden. The manuscript is well written and addresses an important public health issue, particularly given that housing in the post-migration context plays a crucial role in shaping the mental well-being of refugees and asylum seekers. However, there are a few areas where the paper could be strengthened to enhance its contribution to the field, please find specific comments below:

- Response: We appreciate your positive feedback and the time and effort you dedicated to providing input on our manuscript. We have revised our manuscript according to your comments and hope you now find it suitable for publication.

*Introduction*

The introduction provides a strong rationale for the current study, but additional context on how this research builds on and differentiates from existing literature on CMDs among refugees, particularly in the Swedish setting, could be beneficial.

- Response: Thank you for this suggestion. We have added a paragraph about the current literature on CMDs and refugees in Sweden. It now reads as follows: “In the Swedish context, several studies have explored CMDs among different refugee groups, identifying various risk factors such as gender, country of origin, and education [16, 17]. However, no study has specifically examined housing as a risk factor. In terms of studies examining asylum seekers and their housing, several studies have shed light on the conditions within ABO…”

As this study has significant policy implications, adding a brief statement about the current debate around EBO (self-organized housing) and ABO (institutional housing) models could strengthen the introduction and highlight the potential impact of the findings.

- Response: Thank you for this suggestion. We have added a sentence about the current debate in Sweden. It reads as follows: “The political debate primarily focuses on how EBO negatively impacts asylum seekers, particularly through issues like overcrowding, frequent relocations and segregation”

The introduction touches on housing’s role in mental health. Adding a few lines on the pathways linking housing and mental health—such as housing affordability, tenure stability and housing satisfaction—could clarify the conceptual basis for the study.

- Response: Thank you for your input. We have added a sentence to address this and further elaborate on the pathways linking housing and mental health. The revised version is as follows: “Studies also highlight various pathways connecting housing to mental health for asylum seekers and refugees, including factors like insecure tenure, frequent relocations, and the need to share housing due to financial constraints [6]. Other psychosocial factors shown to link housing with mental health included lack of privacy, lack of safety, unfamiliarity or feeling unsafe in neighborhood/location, institutional practices, dissatisfaction with the home, and lack of control [10].”

*Methods*

Line 166: South America was coded as missing (n=20), this needs to be highlighted in the limitations. Was any alternative approach considered?

- Response: Thank you for highlighting this issue. We appreciate your concerns regarding our coding approach. Yes, we conducted sensitivity analyses, as described in the Statistical Analysis section, and we have now clarified this in the paragraph. The revised text now reads “Additionally, sensitivity analyses were performed in which former asylum seekers from South America were included as a separate category within the 'region of origin' variable. These supplementary analyses had no impact on the primary estimates”.

A detailed explanation of why certain sociodemographic factors were considered mediators rather than confounders would clarify the modelling strategy.

- Response: Thanks for the input. According to your suggestion we have clarified why certain factors were considered mediators rather than confounders and we hope this sufficiently describes why these factors might be mediators and as such help explain how or why an exposure affects an outcome. The paragraph now says: “Model 2 additionally adjusted for work status, disposable income, and urbanicity. As studies have indicated [22], these factors can be influenced by housing type; for instance, whether one lives in EBO or ABO may affect employment opportunities or urbanicity. Thus, these factors may serve as mediators in the relationship between the exposure and the outcome, or, expressed differently, may lie within the causal pathway between the exposure and the outcome.”

It would also be helpful to note how missing data were handled for the time-dependent covariates.

- Response: Thanks for your input. We interpret this comment as possibly referring to line 222, which states that GEE "can accommodate subjects with missing values on the independent variables." Therefore, we have clarified in this section that our use of "independent variables" encompasses both time-independent and time-dependent variables, and that GEE manages these in a way that avoids biasing the results. It now reads as follows: “GEE does not require the data to follow a normal distribution and can accommodate subjects with missing values on the independent variables (both time-independent and time-dependent potential confounders and mediators) within the models.”

Additionally, we conducted sensitivity analyses primarily for time-dependent variables with missing data, as described in the last section before the Results, to ensure the accuracy of our estimates. The analyses indicated very little impact on the results. We hope this sufficiently addresses your concerns.

*Results*

This section is well presented.

- Thank you for your positive feedback!

*Discussion*

The policy implications could be expanded. Given that Swedish asylum policy is expected to shift towards more institutionalized accommodation, the authors could discuss how this research might inform future housing policy and mental health support interventions.

- Response: Thank you for your suggestion. In response, we have added a section that elaborates on this topic. It now reads as follows: “When accommodations are assigned based on dispersal and non-choice policies, it is especially important to also discuss how mental health can be promoted during the asylum-seeking period, including the implementation of targeted mental health support interventions that address the unique challenges faced by individuals in these situations. Additionally, involving asylum seekers in the discussion of mental health promotion strategies is crucial to ensure that their needs and experiences are considered.”

Although the manuscript addresses several limitations, an additional note on the potential residual confounding effects of pre-migration trauma, social support, and length of stay in Sweden would provide a more comprehensive view. Expanding on the impact of not including social support measures as potential confounders would be useful, as differences in social support could be significant between ABO and EBO groups.

- Response: Thank you for this suggestion. In response to your feedback and that of the other reviewer, we have made several revisions. For instance, we have added a paragraph in the concluding section of the discussion that expands on the topic of social support. Additionally, we have included an additional note addressing the potential effects of residual confounding. It now reads as follows: “While these are important factors, we have no reason to believe they would vary differentially based on housing type, and no studies support this notion. Nonetheless, it would have further strengthened the validity of our study if we also had adjusted for these factors”

The call for future research could be strengthened by specifying particular areas to explore, such as longitudinal studies that follow asylum seekers in both housing types from arrival through post-asylum periods, or qualitative studies to capture personal experiences within ABO and EBO settings.

- Response: Thank you for highlighting this. We have enhanced the text by including suggestions for future research. It now reads as follows: “To advance these discussions, future research should aim to develop a more comprehensive understanding of the links between housing and mental health for asylum seekers, with a particular focus on contextual factors and the role of social support. Such research should include both qualitative studies that provide in-depth insights into the lived experiences and challenges of asylum seekers as well as quantitative studies – ideally longitudinal surveys – to monitor the health outcomes over time and comparing the effects of different housing types. Ideally, these studies would incorporate a broad set of measures, including social support, pre-migration trauma, mental health status, and the duration of the asylum process.”

We look forward to hearing from you in due time regarding our submission and to respond to any further questions and comments you may have.

Sincerely,

Charlotta van Eggermont Arwidson, PhD student

Department of Neurobiology, Care science and Society, Karolinska Institutet
SE-141 57 Huddinge, Sweden
Email: charlotta.arwidson@ki.se
