# Supplementary material for: Housing during the asylum process and its association with healthcare utilization for common mental disorders among refugees in Sweden: A nationwide cohort study
Source: PLOS Glob Public Health. 2025 May 28;5(5):e0003987. doi: 10.1371/journal.pgph.0003987 (PMC12118870; doi:10.1371/journal.pgph.0003987)
Supplement: Attachment — Submitted filename: Response_to_reviewersCvEArwidsonJan_25.docx [file pgph.0003987.s005.docx]

**Ref: Submission** **PGPH-D-24-01909**

**Response to Reviewers**

Dear Dr. Campbell

We appreciate the opportunity to revise our manuscript, "*Housing during the asylum process and its association with healthcare utilization for common mental disorders among refugees in Sweden: A nationwide cohort study*” and thank you and the reviewers for your valuable feedback.

Below, we provide a point-by-point response outlining the changes made.

We look forward to your response in due course and remain available to address any further questions or comments you may have.

On behalf of all co-authors,

Sincerely,

Charlotta van Eggermont Arwidson, PhD

Comments from the editorial team

Upon further review we are now in a position to grant an exemption on sharing your data as per the journal's requirements. However, even with a data sharing exemption we require additional information to be provided in your Data Availability Statement (DAS) before your manuscript can proceed to publication. Please can you update your DAS ensuring that it includes the following information:

a) Discuss what data you are providing with the paper and the reason why the full underlying data set cannot be made available to other researchers, even upon request.
b) List multiple authors as points of contact and include their contact information (i.e., email address).
c) Provide additional details on how you will ensure persistent or long-term data storage and availability.
d) Specify in your Data Availability statement who will have access to the data and the criteria you will use to consider requests (e.g., the limitations of data sharing as outlined in the IRB approved protocol or patient consent form).
e) Include the following text in your Data Availability statement: “Although the authors cannot make their study’s data publicly available at the time of publication, all authors commit to make the data underlying the findings described in this study fully available without restriction to those who request the data, in compliance with the PLOS Data Availability policy. For data sets involving personally identifiable information or other sensitive data, data sharing is contingent on the data being handled appropriately by the data requester and in accordance with all applicable local requirements.

We understand these comments to mean that the necessary amendments and revisions relate to the data availability statement (DAS), and as per your request, we have included this information in that section.

“The registry data used in this study contains pseudonymized patient-level, medically, sensitive information from nationwide registers. To safeguard the personal integrity of the participating individuals, and following Swedish law—specifically, the Public Access to Information and Secrecy Act—as well as the terms of our ethical approval, such data cannot be made publicly available. Register data can only be made available to someone else after a formal request to access the data. The public rules on publicity and confidentiality necessitate a data-protection assessment, which includes, among other things, that the authority responsible for the research performs a new risk assessment before the data can be disclosed. These rules also apply to requests for data access from researchers not involved in our project, within our organization.

All data will be securely stored on a protected server in de-identified form and retained in accordance with applicable laws and ethical guidelines. Access will be restricted to authorized research personnel involved in the research project, and strict security measures will be in place. Data will be preserved for long-term use within the scope of the study and may be archived or securely deleted as per institutional policies.

Although the authors cannot make their study’s data publicly available at the time of publication, all authors commit to make the data underlying the findings described in this study fully available without restriction to those who request the data, in compliance with the PLOS Data Availability policy. For data sets involving personally identifiable information or other sensitive data, data sharing is contingent on the data being handled appropriately by the data requester and in accordance with all applicable local requirements.

For further questions regarding data availability, contact:

Charlotta van Eggermont Arwidson, [charlotta.arwidson@ki.se](mailto:charlotta.arwidson@ki.se)
Kristina Gottberg, [kristina.gottberg@ki.se](mailto:kristina.gottberg@ki.se)
Petter Tinghög, [petter.tinghog@ki.se](mailto:petter.tinghog@ki.se)”

In addition to this, we interpret the reviewer's comments as indicating that no other changes are necessary to the manuscript. And, after reviewing the reference list with no required modifications, no further revisions have been made to the submission. As a result, the same manuscript as before has been re-uploaded, with no manuscript with tracked-changes.
